# Effect of Different Types of Face Masks on the Ventilatory and Cardiovascular Response to Maximal-Intensity Exercise

**DOI:** 10.3390/biology10100969

**Published:** 2021-09-27

**Authors:** Miguel A. Rojo-Tirado, José A. Benítez-Muñoz, María Alcocer-Ayuga, Víctor M. Alfaro-Magallanes, Nuria Romero-Parra, Ana B. Peinado, Beatriz Rael, Eliane A. Castro, Pedro J. Benito

**Affiliations:** 1 LFE Research Group, Department of Health and Human Performance, Faculty of Physical Activity and Sports Science, Universidad Politécnica de Madrid, 28040 Madrid, Spain; ma.rojo@upm.es (M.A.R.-T.); joseantonio.benitez.munoz@upm.es (J.A.B.-M.); marialcayu@gmail.com (M.A.-A.); vm.alfaro@upm.es (V.M.A.-M.); n.romero@upm.es (N.R.-P.); anabelen.peinado@upm.es (A.B.P.); beanad16@gmail.com (B.R.); eliane.castro@unesp.br (E.A.C.); 2 Department of Physical Education, Faculty of Sciences, Universidade Estadual Paulista (UNESP), Bauru 17033-360, Brazil

**Keywords:** cardiorespiratory fitness, masks, exercise test, athletes, female, exercise tolerance

## Abstract

**Simple Summary:**

The use of facemasks has been part of the non-pharmacological measures for COVID-19 prevention worldwide. However, in many countries their use for sports has not been mandatory due to the additional strain on breathing. It is important to note that the non-use of facemasks in enclosed spaces significantly increases the risk of infection, since breathing and, consequently, the amount of air exhaled during exercise can increase by up to 5 times its value at rest. On the other hand, the choice of mask type could have a major impact on breathing in different types of sports and it is essential to study the different masks available. Thus, the aim of this study was to compare different ways of breathing during exercise using common face masks in young female athletes. Based on our results, the FFP2 mask causes very considerable changes, making breathing difficult, while the Emotion mask has very little impact on the same parameters.

**Abstract:**

The development of new models of face masks makes it necessary to compare their impact on exercise. Therefore, the aim of this work was to compare the cardiopulmonary response to a maximal incremental test, perceived ventilation, exertion, and comfort using FFP2 or Emotion masks in young female athletes. Thirteen healthy sportswomen (22.08 ± 1.75 years) performed a spirometry, and a graded exercise test on a treadmill, with a JAEGER^®^ Vyntus CPX gas analyzer using an ergospirometry mask (ErgoMask) or wearing the FFP2 or the Emotion mask below the ErgoMask, randomized on 3 consecutive days. Also, menstrual cycle status was monitored to avoid possible intrasubject alterations. The results showed lower values for the ErgoMask+FFP2, compared to ErgoMask or ErgoMask+Emotion, in forced vital capacity (3.8 ± 0.2, 4.5 ± 0.2 and 4.1 ± 0.1 l, respectively); forced expiratory volume in 1 s (3.3 ± 0.2, 3.7 ± 0.2 and 3.5 ± 0.1 l); ventilation (40.9 ± 1.5, 50.6 ± 1.5 and 46.9 ± 1.2 l/min); breathing frequency (32.7 ± 1.1, 37.4 ± 1.1 and 35.3 ± 1.4 bpm); VE/VO_2_ (30.5 ± 0.7, 34.6 ± 0.9 and 33.6 ± 0.7); VE/VCO_2_ (32.2 ± 0.6, 36.2 ± 0.9 and 34.4 ± 0.7) and time to exhaustion (492.4 ± 9.7, 521.7 ± 8.6 and 520.1 ± 9.5 s) and higher values in inspiratory time (0.99 ± 0.04, 0.82 ± 0.03 and 0.88 ± 0.03 s). In conclusion, in young healthy female athletes, the Emotion showed better preservation of cardiopulmonary responses than the FFP2.

## 1. Introduction

Due to the SARS-CoV2 pandemic, declared by the World Health Organization on 11 March 2020, all health policies worldwide were forced to implement non-pharmacological interventions, such as social distancing, hand hygiene and the use of face masks, to reduce the transmission of the virus [1]. The recommendation to wear a face mask affects millions of people, even to the athletic population during sports practice in fitness facilities, as recent evidence reports they contribute to reducing virus spread, when the pulmonary ventilation substantially increases [2]. However, although some authors suggest that the use of a face mask can increase carbon dioxide rebreathing, causing hypercapnic hypoxia and, consequently, a decrease in tissue oxygenation, as well as increasing the resistance to inspiration and respiration, thus increasing the work of breathing [3,4,5], other authors suggest that it has minimal impact on physiological function during exercise [6,7] or even no impact on exercise [8,9], generating controversy when using the face mask during exercise, by comparing a wide range of face masks (surgical, FFP2/N95 or cloth masks).

In previous studies, surgical masks (SM) have been compared to FFP2/N95 facepiece respirators in terms of respiratory illness prevention [10,11], suggesting that FFP2/N95 masks are more effective in reducing exposure to viral infections [12]. On the other hand, other research has been carried out with the intention of elucidating the impact of the use of masks at a biological level, such as the possible decrease in tissue oxygenation, previously described. A previous study discovered that, at constant load (steady state exercise), using SM was associated with a significant increase in airway resistance, reduced oxygen uptake, and increased heart rate [13]. Furthermore, some studies compared pulmonary and cardiovascular capacity, at maximal load, using SM and FFP2/N95 in healthy adults [14,15,16], showing that there is a significant reduction in performance (Pmax and VO_2_max) with FFP2/N95, whereas that reduction, although present, is not that consistent with SM. Therefore, according to these studies, medical masks have a marked negative impact on cardiopulmonary capacity that significantly impairs strenuous physical and work activities. Additionally, subjects manifest a progressively higher Borg scale value from no mask < SM < to FFP2/N95, suggestive of a greater dyspnea when masks are worn, at maximal load [14,15]. Likewise, different studies show a perception of discomfort associated with the use of masks [4,15]. Among other perceptions, participants report feeling, at high exercise intensities, respiratory resistance, heat, shortness of breath, claustrophobia and general discomfort [4,17,18].

During the pandemic, research into the creation and development of masks with new fabrics has been significant, resulting in new masks that seek to improve breathability (Pa·cm^−2^) and facial fit, as well as the improvement of oral communication with translucent materials [19], without compromising Bacterial Filtration Efficiency. An example of these innovative products is the Emotion reusable respirators (Texcon y Calidad^®^, Toledo, Spain), that have been recently approved according to the European standard UNE-CWA 17553:2020 [20], providing increased breathability and attempting to counteract the main difficulties reported by society in breathing [21,22,23], such as warmth, anxiety/claustrophobia, alteration of breathing pattern from nasal to oronasal, etc. We believe that research into the use of these new fabrics in the field of sport could contribute to the safe practice of sport without compromising its performance. However, to our knowledge, no scientific literature has analyzed pulmonary and cardiovascular performance with this type of mask, as well as perceived recovery. The main hypothesis of this study was that the FFP2 mask modifies cardiopulmonary function, reducing sports performance, while the Emotion mask does not modify them, thus not reducing work capacity, because they are more breathable. Therefore, the aim of this work was to evaluate whether cardiopulmonary performance, as well as perceived ventilation, exertion, and comfort, are affected using the FFP2 mask or the Emotion mask in young female athletes.

## 2. Materials and Methods

### 2.1. Study Design

The initial sample size was determined through a pilot study with 3 subjects, and a priori power analysis was conducted to determine the final sample size using statistical software (G*Power v. 3.1.9.2, Stuttgart, Germany). A one-way (mask) repeated-measures analysis of variance (ANOVA) was selected as the F test using ventilation and maximum speed test (VE = 101.3 ± 11.9 without mask, 83.7 ± 5,5 for FFP2 and Emotion 96.7 ± 11.2 L/min; Speed = 15.8 ± 0.5 without mask, 15.2 ± 0.4 for FFP2 and 15.7 ± 0.7 Km/h Emotion) as main variables with the following criteria: α = 0.05; (1−β) = 0.8; Effect Size f = 0.30; correlation among repeated measures = 0.5. Calculation via G*Power determined that a sample size of 11 participants was needed to achieve adequate statistical power. In order to finally have this sample, a 20% dropout rate was estimated, so the final sample was calculated as n (1/1-R), where R is the proportion of expected dropouts, so the final sample was 11/0.8 = 14 subjects. The study was carried out on 3 consecutive days, at the same time of the day for each participant. All tests were conducted during the month of June 2021, in the Exercise Physiology Laboratory (Universidad Politécnica de Madrid, Madrid, Spain), with environmental conditions of 26.6 ± 1.5 °C temperature, 29.2 ± 6.7 % relative humidity and 721 ± 25 mmHg atmospheric pressure. The participants were scheduled according to their menstrual cycle, as detailed below. They were asked to eat a similar diet and to avoid caffeine on all three days of the study. To ensure rest, they were not allowed to engage in physical activity 24 h before the tests and the hours of sleep were checked. On the first day of the study, prior to the start of testing, the participants were required to have a negative antigen test result for COVID-19. Then, a medical evaluation was thoroughly performed, as well as an evaluation of body composition, a resting electrocardiogram and two baseline spirometries wearing a respiratory mouthpiece or an ergospirometry mask (ErgoMask, Jaeger-CareFusion, Hoechberg, Germany). Below, a graded exercise test (GXT) with the ErgoMask until exhaustion followed by a confirmatory test was carried out. On days 2 and 3, both a basal spirometry and a GXT were performed wearing the FFP2 mask (FFP2) or the Emotion mask below the ErgoMask (ErgoMask+FFP2 and ErgoMask+Emotion, respectively). The order of the ErgoMask+FFP2 and ErgoMask+Emotion was randomly and counterbalanced assigned for days 2 and 3.

The recruitment process consisted of several advertisement campaigns covering a wide variety of media. Interested people contacted us, where an online questionnaire was filled out to check inclusion criteria. Finally, a total of 36 potential participants who met the inclusion criteria were recruited. They were informed about the nature of the study, and those who met the inclusion criteria were invited to participate in the study. Finally, 13 healthy sportswomen were involved in the study (22.08 ± 1.75 years, 59.06 ± 5.59 kg, 1.66 ± 0.06 m and body fat of 21.18 ± 4.73 %). The inclusion criteria were: (1) being female, (2) aged 18 to 30 years, (3) with regular menstrual cycles or using hormonal contraceptives, (4) who trained any endurance sport (athletics, triathlon, etc.) at least 3 h per week, to ensure that its protocols were maximized, (5) not having a respiratory disease and (6) who were available to come to the laboratory 3 consecutive days. The reasons for exclusion were as follows: (1) 19 persons did not meet the inclusion criteria, (2) 1 person dropped out, (3) 1 person had an unconventional respiratory pattern and (4) 2 persons were asthmatics not previously diagnosed. The study was conducted in accordance with the Declaration of Helsinki, revised in Fortaleza in 2013, and was approved by the Ethical Committee of the Universidad Politécnica de Madrid (reference number: 2021-030, 2021-031, 2021-032). Written informed consent was obtained from all the participants.

### 2.2. Face Masks

Two different face masks were compared in this study. On the one hand was the FFP2 protective face mask (TEDISA, Textil Distribuidora, S.A., Alcorcón, Madrid, Spain, https://www.tedisaprotection.es/s1597, accessed on 15 September 2021) and the Emotion mask (Texcon y Calidad^®^, Toledo, Spain, https://www.mascarillasemotion.com/emotion/), made with a 100% polyamide main fabric and a 65% polyester–35% cotton secondary fabric, and tested by the AMSLAB (Applied Mass Spectometry Laboratory, Lugo, Spain) laboratory [19]. The ErgoMask was placed over the FFP2 and the Emotion masks. The ErgoMask was properly adjusted by the technicians to avoid air leaks, and all possible gaps were fixed with adhesive tape. The adjustment of the ErgoMask was confirmed if the participant was unable to exhale while closing the exit hole of the mask by the technician.

### 2.3. Menstrual Cycle Control

Menstrual cycle status was monitored to avoid possible intrasubject alterations in the variables explored, since there may be cardiorespiratory variations throughout the different phases of the menstrual and oral contraceptive cycles [24,25]. Therefore, the aim of this control was to evaluate participants under similar sex hormone status during the three consecutive testing days. Based on the current considerations for research in sport and exercise science with women as participants [26], the first day of the menstrual cycle started with the onset of menstrual bleeding. Furthermore, eumenorrheic participants performed a urinary ovulation test (Ellatest, Alicante, Spain) to detect the luteinizing hormone surge occurring 24–36 h before ovulation from 3 days before the predicted ovulation day by the calendar-based counting method until a positive test was recorded, as previously indicated [26,27]. In accordance with this, participants with eumenorrheic cycles were evaluated during the mid-follicular phase (day 6 to 10 of the cycle) or during the mid-luteal phase (+5 to +9 days after confirmed ovulation). Moreover, to further discard ovulation during the evaluation period, participants also performed a urinary ovulation test from two days before starting the evaluation period to two days after finishing it. On the other hand, monophasic oral contraceptive users were evaluated during the second half of the 21 days of pill consumption (active pill phase) as sex hormone levels are fairly stable during these days [26]. Moreover, all participants ingested the oral contraceptive pill upon awakening in the morning to avoid the hormonal surge produced in the hours following ingestion, and pill consumption was verbally confirmed with the participants daily from two days before starting the evaluation period until finishing it.

### 2.4. Body Composition

On the first day of evaluation, body weight was measured with a scale (Lafayette Instruments Company, Lafayette, IN, USA) and height with a stadiometer (Holtain Limited, Crymych, UK). Then, a dual-energy X-ray absorptiometry (DXA) was performed on each participant to determine body composition. Calibration and evaluation procedures were performed by recommendations of the manufacturer and certified technicians. To help the participants move as little as possible during the test, they were helped by holding their legs by the ankles with a strap. It was carried out using a GE Lunar Prodigy apparatus (GE Healthcare, Madison, WI, USA), and scan analyses were performed using GE Encore 2002 software v 6.10.029.

### 2.5. Spirometry

A spirometry was performed for each participant on calibrated JAEGER^®^ Vyntus CPX (Jaeger-CareFusion, Hoechberg, Germany) equipment. The technician explained and demonstrated the two different tests to be performed exhaustively, the Force Vital Capacity (FVC) and the Maximum Voluntary Ventilation (MVV). The Force Vital Capacity (FVC), Force expiratory volume in 1 s (FEV1), Tiffenau index and Maximum Voluntary Ventilation) were evaluated from this test. On the first visit, the FVC and the MVV were carried out with the respiratory mouthpiece placed in the mouth and the clamp on the nose of the participant (Mouthpiece) and, subsequently, wearing the ErgoMask. On the second and the third visit, the FVC and the MVV tests were performed wearing ErgoMask+FFP2 or ErgoMask+Emotion. The FVC and the MVV were performed according to the current guidelines [28]. Both tests were carried out with a gas analyzer, JAEGER^®^ Vyntus CPX (Jaeger-CareFusion, Hoechberg, Germany), previously calibrated according to the manufacturer.

### 2.6. Graded Exercise Test

During the first visit, the GXT was performed wearing the ErgoMask only, and wearing the ErgoMask+FFP2 or ErgoMask+Emotion on the second and the third visit. The order of the face masks on the second or third day was randomly assigned. Participants were informed about the GXT and the safety criteria that they should follow in case of physical discomfort. The GXT was conducted on a computerized treadmill (H/P/COSMOS 3PW 4.0, H/P/Cosmos Sports & Medical, Nussdorf-Traunstein, Germany). Volume and composition of expired gas was analyzed with a JAEGER^®^ Vyntus CPX gas analyzer (Jaeger-CareFusion, Hoechberg, Germany), previously calibrated according to the manufacturer, which validity and reliability has been demonstrated (coefficient of variation of respiratory quotient below 0.5%) [29]. Heart rate (HR) was continuously monitored with the pulsometer Polar RS800sd (Polar Electro Oy, Kempele, Finland).

**Figure 1:**
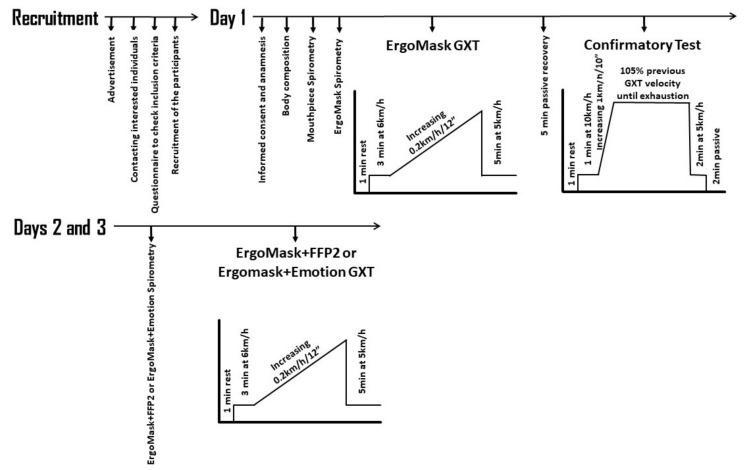
Study design. The order of the ErgoMask+FFP2 and ErgoMask+Emotion on days 2 and 3 was randomly and counterbalanced assigned. ErgoMask GXT: Graded exercise test performed wearing the ergospirometry mask; ErgoMask+FFP2 Spirometry: Spirometry performed wearing the FFP2 mask below the ergospirometry mask; ErgoMask+Emotion Spirometry: Spirometry performed wearing the Emotion mask below the ergospirometry mask; ErgoMask+FFP2 GXT: Graded exercise test performed wearing the FFP2 mask below the ergospirometry mask; ErgoMask+Emotion GXT: Graded exercise test performed wearing the Emotion mask below the ergospirometry mask. Participants.

The GXT was performed at 1% incline to simulate resistance from overground running. The GXT started with a warm-up at 6 km/h for 3 min. Then, the incremental phase started at 8 km/h, and running velocity was increased by 0.2 km/h every 12 s (1 km/h/min). The incremental phase finished when the participant was unable to maintain the velocity of the treadmill. The test ended after an additional 5 min of active recovery at 5 km/h on the treadmill. On the first day, a confirmatory test [30] was performed after 5 min of passive recovery following the GXT. The confirmatory test consisted of 1 min at 10 km/h followed by increments of 1 km/h every 10 s until reaching 105% of the maximal velocity achieved in the previous GXT (see Figure 1). The confirmatory test finished when the sub-jects could not maintain the velocity of the treadmill.

The following cardiorespiratory variables were evaluated from the GXT: ventilation (VE), oxygen consumption (VO_2_), carbon dioxide production (VCO_2_), respiratory exchange ratio (RER), VE/VO_2_, VE/VCO_2_, tidal volume (TV), breathing frequency, inspiratory time (Tins), expiratory time (Texp) and HR. In addition, the performance variables time to exhaustion and speed were evaluated.

VO_2_max was considered achieved if two of the three following criteria were reached: heart rate (HR) ≥95% of the theoretical maximum HR calculated as 220-age, RER ≥1.10 and VO_2_ plateau despite increasing the exercise intensity [31]. The VO_2_max was stablished as the highest value obtained during the GXT. A difference of less than 2% in the maximum oxygen consumption value between the GXT and the confirmatory test was used as a criterion for determining a true VO_2_max [32]. The first and the second ventilatory threshold (VT1 and VT2, respectively) were set at the point of maximum agreement of the most common methods of assessment published previously [33]. Briefly, VT1 was calculated: (1) according to the V-slope method, whereby VT1 is the break point of the VCO_2_–VO_2_ relationship (VCO_2_ is carbon dioxide production and VO_2_ is oxygen consumption); (2) as the first exponential increment in ventilation (VE); and (3) as the first rise in the VE/VO_2_ relationship without increments in the VE/VCO_2_ relationship. VT2 was determined: (1) as the second rise in ventilation; (2) as the intensity that accompanied a second rise in the VE/VO_2_ relationship with a concurrent rise in the VE/VCO_2_ relationship. All tests were evaluated by 2 researchers, and a third researcher was asked in case of controversy.

### 2.7. Subjective Scales

Participants were administered three different subjective scales during rest (rest), in the warm-up (warm-up), at the end of the test (max) and after five minutes of recovery (300 s rec). Perceived ventilation was measured with a visual analog scale (VAS VE) of 0–10 (from 0: I cannot breathe; to 10; I breathe perfectly), the rate of perceived exertion (RPE) with a visual scale of 0–10 (from 0: not at all intense; to 10; extremely intense) [14,15], and degree of mask comfort with a visual analog scale of 0–10 (from 0: very comfortable; to 10; extremely uncomfortable) [4,15,17,18]. Before the start of the tests, all scales were explained to the participants, so that they were familiar with them, and all possible doubts were answered to ensure the correct application of these scales during the project.

### 2.8. Statistical Analysis

All values are expressed as mean ± standard error of the mean (SEM). Confidence limits (95%) are shown in tables. The Shapiro–Wilk test was used to check the normal distribution of the data. A one-way ANOVA for repeated measures was performed to analyze the influence of the different masks (Mouthpiece, ErgoMask, ErgoMask+FFP2 and ErgoMask+Emotion) on spirometry variables. A two-way ANOVA for repeated measures (3 × 7) was performed to analyze the effect of the different masks (ErgoMask, ErgoMask+FFP2 and ErgoMask+Emotion) and the time of measurement (rest, warm-up, VT1, VT2, maximal, 120 s of recovery and 300 s of recovery) on cardiorespiratory variables. A two-way ANOVA for repeated measures (3 × 3) was performed to analyze the effect of the different masks and time of measurement (VT1, VT2 and max) on performance variables. Finally, a two-way ANOVA for repeated measures (3 × 4) was used to analyze the effect of the different masks and time of measurement (rest, warm-up, max and 300s rec) on subjective scales. Mauchly’s sphericity test was carried out to evaluate whether the sphericity assumption of the variances was violated, in which case the Huynh–Feldt correction was applied. Bonferroni post hoc tests were conducted where significant differences were found in any of the analyzed factors. ANOVA effect size was calculated by partial eta-squared (η_p_^2^) and small, moderate and large effect corresponded to values equal to or greater than 0.001, 0.059 and 0.138, respectively [34]. Pairwise comparisons effect size was calculated by Cohen’s considering d < 0.5 as small, d < 0.8 as moderate and d > 0.8 as large [35]. Data were analyzed using the SPSS statistic software, version 25.0, for Windows (IBM Corporation; Armonk, NY, USA). The significance level was set at *p* < 0.05.

## 3. Results

The spirometry results are shown in Table 1. We observed a significant main effect of mask on FVC (F_2,15_ = 9.795, *p* = 0.002, η^2^ = 0.495), FEV1 (F_3,30_ = 12.376, *p* < 0.001, η^2^ = 0.553) and MVV (F_2,19_ = 16.670, *p* < 0.001, η^2^ = 0.625). Pairwise comparisons are shown in Table 1. No significant effect of mask was observed on TIFF (F_3,30_ = 1.703, *p* = 0.187, η^2^ = 0.146).

The main effects of masks on ergospirometry variables (average of the seven times of measurement of the GXT) and pairwise comparisons are shown in Table 2. A significant effect of masks was observed for VE, VO_2_, VO_2_/kg, VE/VO_2_, VE/VCO_2_, Time to exhaustion, Breathing frequency, Tins, Texp and VAS VE. The ErgoMask+Emotion has shown higher VE, VE/VO_2_, VE/VCO_2_, Breathing frequency and Time to exhaustion compared to the ErgoMask+FFP2, while Tins was lower with the ErgoMask+Emotion. When comparing the ErgoMask with the ErgoMask+FFP2, higher VE, VO_2_, VCO_2_ and VAS VE were observed.

**Figure 2:**
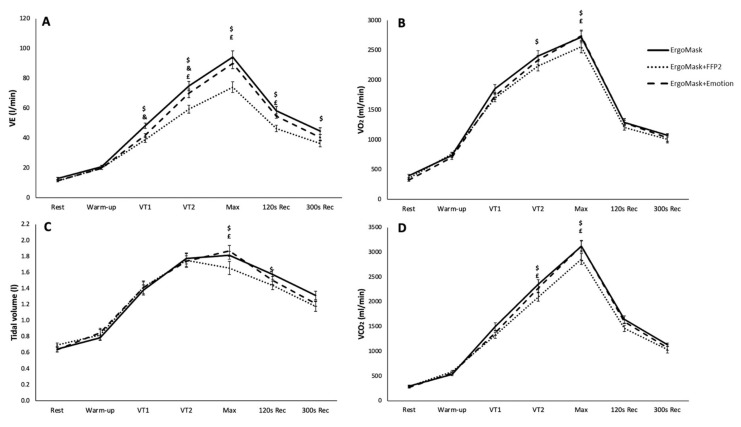
Ventilation (VE) (**A**), Oxygen consumption (VO_2_) (**B**), Tidal volume (**C**) and Carbon dioxide output (VCO_2_) (**D**) throughout the graded exercise test (Mean ± SEM). ErgoMask: Ergospirometry Mask; ErgoMask+FFP2: Ergospirometry Mask + FFP2; ErgoMask+Emotion: Ergospirometry Mask + Emotion; VT1: Ventilatory threshold 1; VT2: Ventilatory threshold 2; Max: Maximal; 120 s Rec: 120 s of recovery; 300 s Rec: 300 s of recovery. $ Significant differences between ErgoMask and ErgoMask+FFP2 (*p* ≤ 0.05); & Significant differences between ErgoMask and ErgoMask+Emotion (*p* ≤ 0.05); £ Significant differences between ErgoMask+FFP2 and ErgoMask+Emotion (*p* ≤ 0.05).

Interaction effects between mask and time are shown in Figure 2. A significant interaction was observed for VE (F_5,58_ = 5.042, *p* < 0.001, η^2^ = 0.296), VO_2_ (F_8,55_ = 2.179, *p* = 0.037, η^2^ = 0.154), VCO_2_ (F_12,143_ = 3.496, *p* < 0.001, η^2^ = 0.226) and Tidal volume (F_8,58_ = 2.176, *p* = 0.032, η^2^ = 0.154). At VT1, the ErgoMask showed a higher VE (Figure 2A) compared to ErgoMask+FFP2 and ErgoMask+Emotion, but from VT2 to 120 s Rec, both ErgoMask and ErgoMask+Emotion showed higher values in comparison to ErgoMask+FFP2. Likewise, VO_2_ (Figure 2B), VCO_2_ (Figure 2D) and Tidal volume (Figure 2C) were also higher at Max with the ErgoMask and ErgoMask+Emotion than with ErgoMask+FFP2. No other significant interactions were observed for the rest of the ergospirometry variables (RER, VE/VO_2_, VE/VCO_2_, Breathing frequency, Tins, Texp, HR, Time to exhaustion, Speed, VAS VE and RPE; see Supplementary Material Table S1).

Finally, no effects of mask or interaction between mask and time were observed for the different scales administered to rate aspects related to mask comfort such as humidity, heat, smell, breathing impairment, fatigue, tightness, or suitability (data not shown).

## 4. Discussion

When, by the end of spring 2020, the general population worldwide was encouraged to use face masks to prevent SARS-CoV-2 from spreading [1], the common feeling was that face masks would impair everyday-life performance, and that they would make it impossible to practice sport. However, reality has proven otherwise. Anyway, there is still an important concern about the safety of face masks in high-intensity training, and how they can affect performance. We chose to study an athletic population, as previously done by Egger et al. [16], to have a specific approach to maximal-intensity exercise, and we chose to do it on a treadmill rather than a bicycle ergometer, in contrast to all recent studies [14,15,16], to better mimic their everyday athletic gesture. This study ratifies that face masks, both FFP2 and Emotion, are safe for healthy, athletic people to practice sport with, even at high intensities. Regarding performance, several previous studies [14,15,16] have reported a significant reduction in cardiopulmonary and global performance with the use of face masks, both surgical and FFP2. This is the first study to analyze physical response to the use of a specially developed face mask, Emotion reusable respirator, compared to no mask and with the use of FFP2. Interestingly, cardiopulmonary performance was globally very similar with no mask and the use of Emotion, this way improving previously reported results from cloth and surgical masks [4,13,14,15,16]. On the other side, it was significantly lower with FFP2, confirming previously reported findings [14,15].

As previously mentioned, and in the first place, regarding safety, no adverse event was reported, neither hemodynamic, nor electrical, nor clinical.

With respect to pulmonary function, resting parameters already show differences between FFP2 and no mask or Emotion, as in the basal spirometry there is a significant worsening in FVC and FEV1 with the FFP2, though no such difference is seen with the Emotion. There is no difference, however, in the FEV1/FVC index, suggesting that, at rest, FFP2 (but not Emotion) induces airway resistance to inhalation, as has already been demonstrated by other authors [5,14,15], but no obstruction.

However, during exercise, the use of Emotion does slightly but significantly alter the breathing pattern, because, from VT1 to peak exercise, ventilation decreases (between 6.5 and 4.2 L/min–13.5 and 4.5%) compared to no mask. This decrease responds to the reduction of breathing frequency (Bf) because tidal volume remains the same as no mask all through the test. It is noticeable how this difference is progressively lower as intensity goes up, confirming the possibility of adaptation to the use of the mask. In contrast, with the FFP2 mask, this adaptation is not possible: VE starts decreasing at VT1 (by a mean of 9.3 L/min, 19.3%), and continues to decrease until peak exercise to a mean of 20.1 L/min (22.3% of maximum VE reached with no mask). At VT1 and VT2, tidal volume does not change compared to no mask and Emotion, so this reduction responds only to the decrease in Bf, which, at the same time, can be explained by the increase of both Tins and Texp. Maximum TV is reached at VT2: from VT2 to peak exercise, not only does TV not increase, but it decreases substantially, with a much steeper augmentation of Bf compared to no mask and Emotion, probably because of respiratory muscle fatigue, which induces breathing to be shallower but faster. Physiologically, from VT1 on, the increase of ventilation is necessary to prevent acidosis, but when reaching VT2, this increase is mandatory, as acidosis is already established, to prevent pH from going dangerously down [36]. As it is, the observed change in the breathing pattern allows the participants to keep up with the required VE with a slightly lesser effort.

These objective findings were completed with the Visual Analogic Scale for ventilatory effort perception (VAS VE), as subjects reported significantly higher perceived ventilatory effort with FFP2 in all stages of the test, as previously reported by other past studies [14,15,16,17,18].

Concerning cardiovascular function and ventilatory efficiency, it is important to highlight that we did not find differences between VO_2_max with the use of Emotion compared to no mask (46.36 mL/min/kg vs. 46.07 mL/min/kg). On the other side, there is a significant, though not as high as probably expected, by terms of comparison to previous data [14,15,16], decrease of VO_2_max with the use of FFP2 (by a mean of 2.9 mL/kg/min, 6.3% of VO_2_max with no mask, whereas Mapelli et al. [14] reported a reduction of 10%, Fikenzer et al. [15] of 13% and Egger et al. [16] of 19%). Without any obstacle that can potentially impair ventilation, VCO_2_ output is comparable to CO_2_ production in cellular respiration during exercise. However, as we have already seen, both masks are an obstacle impairing ventilation (although to different extents), so VCO_2_ output cannot be assumed to be equivalent to CO_2_ production until proven otherwise. Provided one exerting body should not change CO_2_ production from one test to the other (meaning, if testing protocols are the same, cellular respiration processes should also be the same), VE, and, thus, VE/VCO_2_, form the meaningful parameter to understand VCO_2_ output. There were no differences between no mask and Emotion in VCO_2_ all through the test (suggesting that the obstacle Emotion produces in ventilation is not enough to diminish VCO_2_ output), whereas there is a significant decrease with the use of FFP2, but only at VT2 and peak exercise. Nevertheless, this decrease is proportionally not as high as the decrease in ventilation, as beautifully shown by the difference in VE/VCO_2_, which is significantly lower with the FFP2 (25.7, versus 27.8 with Emotion and 28.9 with no mask), thus suggesting that, as long as we are referring to healthy individuals, face masks may trigger the improvement of ventilatory efficiency to keep up with exercise requirements.

Heart rate (HR) is one of the parameters that modulates cardiac output, along with stroke volume. They are both mediated by autonomic control, due to neurohormonal response to tissue and myocardial oxygen demand. This, and the fact that tests were carried out over three consecutive days, explain why heart rate has demonstrated to be the only submaximal parameter to remain unaltered despite both the masks, all through the seven times of measurement of the tests. This is an important finding concerning people who practice endurance sports and adjust the training intensity according to HR in both VT1 and/or VT2: the use of face masks does not modify VT1, VT2 or maximal heart rate. Arterial pressure during rest, at peak exercise and at recovery also shows no difference between no mask and both face masks.

Regarding work capacity, achieved peak speed was very similar in all tests; there is a mild decrease with the use of FFP2 (by 0.24 km/h, to a mean speed of 15 km/h), which is not statistically significant. Nonetheless, as a more sensitive parameter, time to exhaustion was significantly longer with Emotion than FFP2, and even longer (though not significantly) than that with no mask. This last finding can be justified as the learning effect of performing three maximal exercise tests over three consecutive days, the first one always being the one with no mask. However, this learning effect can only be benefited by the Emotion and not FFP2, as tests with the face masks were randomized, and thus, this effect counterbalanced. In any case, more importantly, the most meaningful finding is the shortest time (by almost one minute compared to no mask, 42 s compared to Emotion) and lower speed (1 km/h compared to no mask, 0.76 km/h compared to Emotion) to reach VT2 with the FFP2. To this, there a physiological explanation: as VCO_2_ output is impaired by the use of the mask, the excess of CO_2_ that is produced from VT1 on (when HCO_3_^-^ buffers the excess of lactate in blood) [37] cannot be properly exhaled, so compensation mechanisms fail and metabolic acidosis appears earlier, and so VT2 overcomes sooner, being, this way, shifted to the left. No such differences are statistically significant with Emotion compared to no mask.

This is the first study conducted on women. In addition to that, one of the main strengths of this study is to investigate, not only healthy, but also athletic individuals. Thereby, we made sure we started from the basis of the best possible physical conditions, that VT2 could be easily surpassed, and that maximum oxygen uptake could be reached. As previously mentioned, VT2 has proven to be the point in which cardiopulmonary performance trends go down when wearing FFP2, so the main strength of this study is the ability to identify this point and explain the physiological mechanisms to which such impairment responds.

However, this study is not exempt of limitations. As the study was conducted only on healthy, young, athletic women, these results should be extrapolated to the general population with caution. Despite having checked the tightness of the masks, it is not possible to be sure that there were no leaks. We did place a saturometer in the ear of the participants, but we were only able to assess reliable SpO_2_ measures from immediate recovery onwards, probably due to peripheral vasoconstriction induced by high-intensity exercise. The lack of data on blood testing impedes giving further explanations to some of the findings, like, for example, if the fact that VT2 overcomes sooner affects the total amount of lactate produced. Further studies should be conducted to assess safety and performance with face masks on the general population, especially sedentary people and patients with cardiovascular, pulmonary and neuromuscular disease.

As practical application, this study not only encourages people to continue practicing sport despite the use of face masks, that will probably be mandatory in sports facilities for some time, but also shows that the use of Emotion allows individuals to reach maximal intensities, with similar workload and heart rate at both of each threshold, and with minimal impact on respiratory effort. The FPP2 mask, however, produces significant ventilatory impairment at high intensities, so it is not recommended (though neither totally discouraged, as long as we are assessing healthy individuals) to use an FFP2 mask to train at workloads above VT2. Further research can probably provide knowledge on the mechanisms that produce the decrease of VO_2_max with the use of FFP2, thus enabling professionals to give specific recommendations to patients.

## 5. Conclusions

For sport practice, the use of the Emotion mask has shown, in most of the ergospirometric variables, a very similar behavior to not wearing a mask. In contrast, using the FFP2 mask, the workload above VT2 was lower and there was a significant ventilatory compromise at high intensities.

## Figures and Tables

**Table 1:** Spirometry results (*n* = 11). Shown as mean ± standard error of the mean (SEM) and 95% confidence limit.

	Spirometry Mouthpiece		ErgoMask		ErgoMask+FFP2		ErgoMask+Emotion	
FVC (l)	4.2 ± 0.2	(3.8–4.7)	4.5 ± 0.2	(3.9–5.0)	3.8 ± 0.2 ^§,^*	(3.5–4.2)	4.1 ± 0.1 **^#^**	(3.8–4.5)
FEV_1_ (l)	3.7 ± 0.2	(3.3–4.1)	3.7 ± 0.2	(3.3–4.2)	3.3 ± 0.2 ^§,^*	(2.9–3.6)	3.5 ± 0.1 **^#^**	(3.2–3.9)
TIFF (%)	86.7 ± 2.1	(82.1–91.3)	83.7 ± 2.9	(77.3–90.2)	85.5 ± 2	(81.0–90.0)	85.4 ± 1.7	(81.6–89.2)
MVV (l/min)	122.5 ± 5.3	(110.8–134.2)	117.9 ± 6.3	(103.8–132.0)	99.4 ± 4.8 ^§^	(88.7–110.1)	127.4 ± 5.0 **^#^**	(116.2–138.7)

Note. ErgoMask: Ergospirometry Mask; ErgoMask+FFP2: Ergospirometry Mask+FFP2; ErgoMask+Emotion: Ergospirometry Mask+Emotion; FVC: Forced vital capacity; FEV1: Forced expiratory volume in 1 s; TIFF: Tiffenau index; MVV: Maximal voluntary ventilation. ^§^ Significant differences with Spirometry Mouthpiece (*p* < 0.05); * Significant differences with ErgoMask (*p* < 0.05); ^#^ Significant differences with ErgoMask+FFP2 (*p* < 0.05).

**Table 2:** Average values for ergospirometry variables and subjective scales (*n* = 13). Shown as mean ± standard error of the mean (SEM) and 95% confidence limit.

	ErgoMask		ErgoMask+FFP2		ErgoMask+Emotion		F	*p*	η^2^
VE (l/min)	50.6 ± 1.5	(47.4–54.9)	40.9 ± 1.5 *^1^	(37.6–44.2)	46.9 ± 1.2 *^2,#1^	(44.2–49.5)	33.887	<0.001	0.738
VO_2_ (ml/min)	1497 ± 48	(1392–1602)	1404 ± 46 *^3^	(1301–1506)	1451 ± 38	(1368–1534)	6.189	0.007	0.340
VO_2_/kg (ml/min/kg)	25.4 ± 0.6	(24.0–26.8)	23.7 ± 0.6 *^4^	(22.5–24.9)	24.6 ± 0.3	(23.9–25.2)	6.517	0.005	0.352
VCO_2_ (ml/min)	1512 ± 50	(1401–1621)	1310 ± 47 *^5^	(1276–1482)	1469 ± 38	(1385–1551)	6.854	0.004	0.364
RER	0.96 ± 0.01	(0.94–0.99)	0.97 ± 0.02	(0.93–1.00)	0.98 ± 0.01	(0.96–1.00)	0.468	0.632	0.038
VE/VO_2_	34.6 ± 0.9	(32.7–36.5)	30.5 ± 0.7 *^6^	(29.0–32.1)	33.6 ± 0.7 ^#2^	(32.1–35.1)	29.901	<0.001	0.714
VE/VCO_2_	36.2 ± 0.9	(34.3–38.2)	32.2 ± 0.6 *^7^	(30.8–33.6)	34.4 ± 0.7 *^8,#3^	(32.9–35.8)	30.657	<0.001	0.719
TV (l)	1.33 ± 0.03	(1.26–1.40)	1.28 ± 0.04	(1.18–1.38)	1.32 ± 0.05	(1.21–1.42)	1.551	0.233	0.114
Breathing frequency (bpm)	37.4 ± 1.1	(35.0–39.8)	32.7 ± 1.1 *^9^	(30.2–35.2)	35.3 ± 1.4 ^#4^	(32.2–38.4)	14.717	<0.001	0.551
Tins (s)	0.82 ± 0.03	(0.75–0.89)	0.99 ± 0.04 *^10^	(0.90–1.07)	0.88 ± 0.03 ^#5^	(0.81–0.96)	10.958	<0.001	0.477
Texp (s)	1.01 ± 0.05	(0.90–1.12)	1.15 ± 0.06 *^11^	(1.03–1.28)	1.05 ± 0.05	(0.94–1.16)	5.155	0.014	0.300
HR (bpm)	132 ± 2	(126.8–137.1)	135 ± 3	(127.6–131.5)	132 ± 2	(127.0–136.2)	0.912	0.415	0.071
Time to exhaustion (s)	521.7 ± 8.6	(502.9–540.5)	492.4 ± 9.7	(471.3–513.5)	520.1 ± 9.5 ^#6^	(499.3–540.8)	6.499	0.006	0.351
Speed (km/h)	12.4 ± 0.2	(12.1–12.7)	11.9 ± 0.1	(11.6–12.2)	12.3 ± 0.2 ^#7^	(11.9–12.7)	4.430	0.023	0.270
VAS VE (0–10)	7.5 ± 0.3	(6.7–8.3)	5.5 ± 0.7 *^12^	(3.4–6.4)	6.8 ± 0.4	(6.0–5.7)	6.007	0.009	0.375
RPE (0–10)	2.9 ± 0.3	(2.3–3.5)	3.1 ± 0.2	(2.6–3.5)	3.0 ± 0.2	(2.6–3.5)	0.496	0.616	0.047

Note. ErgoMask: Ergospirometry Mask; ErgoMask+FFP2: Ergospirometry Mask+FFP2; ErgoMask+Emotion: Ergospirometry Mask+Emotion; VE: Ventilation; VO_2_: Oxygen consumption; VO_2_/Kg: Oxygen consumption relative to body mass; VCO_2_: Carbon dioxide production; RER: Respiratory exchange ratio; Tins: Inspiratory time; Texp: Expiratory time; HR: Heart rate; VAS VE: Ventilation visual analogue scale; RPE: Rate of perceived exertion; * Comparing with ErgoMask: *^1^ *p* < 0001, d = 1.79; *^2^ *p* < 0.001, d = 0.76; *^3^ *p* = 0.014, d = 0.55; *^4^ *p* = 0.013, d = 0.79; *^5^ *p* = 0.025, d = 1.15; *^6^ *p* < 0.001, d = 1.41; *^7^ *p* < 0.00,1 d = 1.45; *^8^ *p* = 0.04, d = 0.62; *^9^ = *p*<0.001, d = 1.19; *^10^ *p* = 0.002, d = 1.33; *^11^ *p* = 0.038, d = 0.70; *^12^ *p* = 0.052, d = 1.03. ^#^ Comparing with ErgoMask+FFP2: ^#1^ *p* = 0.002, d = 1.23; ^#2^ *p* < 0.001, d = 1.23; ^#3^ *p* < 0.001, d = 0.94; ^#4^ *p* = 0.045, d = 0.57; ^#5^ *p* = 0.018, d = 0.86; ^#6^ *p* = 0.006, d = 0.8; ^#7^ *p* = 0.053, d = 0.7.

## Data Availability

Data sharing not applicable.

## Supplementary Materials

The following are available online at https://www.mdpi.com/article/10.3390/biology10100969/s1, Table S1: Effect of interaction for ergospirometry variables and subjective scales expressed as mean ± standard error of the mean (SEM).
