# Supplementary material for: Effect of Different Types of Face Masks on the Ventilatory and Cardiovascular Response to Maximal-Intensity Exercise
Source: Biology (Basel). 2021 Sep 27;10(10):969. doi: 10.3390/biology10100969 (PMC8533493; doi:10.3390/biology10100969)
Supplement: Supplementary file 1 [file biology-10-00969-s001.zip › biology-1347982-supplementary.pdf]

Table S1. Effect of interaction for ergospirometry variables and subjective scales expressed as mean ± standard error of the mean (SEM).

		Rest	Warm-up	VT1	VT2	Max	120s Rec	300s Rec	F	p	η^2
RER	**ErgoMask**	0.76 ± 0.01	0.72 ± 0.02	0.81 ± 0.02	0.98 ± 0.02	1.15 ± 0.01	1.28 ± 0.02	1.05 ± 0.01	1.838	0.189	0.133
	**ErgoMask+FFP2**	0.82 ± 0.02	0.78 ± 0.02	0.78 ± 0.02	0.94 ± 0.02	1.23 ± 0.11	1.21 ± 0.02	1.02 ± 0.01			
	**ErgoMask+Emotion**	0.85 ± 0.02	0.80 ± 0.02	0.79 ± 0.02	0.97 ± 0.01	1.15 ± 0.01	1.25 ± 0.03	1.05 ± 0.02			
VE/VO_2	**ErgoMask**	33.0 ± 1.0	28.5 ± 1.0	26.2 ± 0.5	31.3 ± 1.0	34.9 ± 1.5	45.2 ± 1.7	41.6 ± 1.5	1.898	0.065	0.137
	**ErgoMask+FFP2**	31.5 ± 1.0	26.3 ± 0.8	23.0 ± 0.7	26.6 ± 1.0	29.0 ± 1.0	38.5 ± 1.1	36.4 ± 1.0			
	**ErgoMask+Emotion**	35.2 ± 1.1	28.3 ± 0.8	24.3 ± 0.8	30.0 ± 0.9	33.1 ± 1.2	42.9 ± 1.6	39.8 ± 1.5			
VE/VCO_2	**ErgoMask**	43.7 ± 1.3	39.4 ± 1.2	32.5 ± 0.7	32.0 ± 0.9	30.3 ± 1.1	35.4 ± 1.1	39.5 ± 1.2	0.769	0.681	0.060
	**ErgoMask+FFP2**	38.5 ± 1.4	34.0 ± 0.9	29.5 ± 0.7	28.4 ± 0.7	26.1 ± 0.8	31.8 ± 0.5	35.7 ± 1.1			
	**ErgoMask+Emotion**	41.5 ± 1.2	35.7 ± 0.9	30.7 ± 0.9	30.9 ± 0.8	28.9 ± 1.0	34.3 ± 0.7	37.9 ± 1.2			
Breathing frequency (bpm)	**ErgoMask**	21.3 ± 1.2	28.2 ± 1.2	37.8 ± 1.8	44.8 ± 2.0	55.1 ± 2.1	38.5 ± 1.7	36.2 ± 2.0	1.025	0.419	0.079
	**ErgoMask+FFP2**	18.3 ± 1.1	24.4 ± 1.5	31.3 ± 2.1	37.5 ± 2.1	50.4 ± 2.0	33.9 ± 1.4	33.0 ± 1.4			
	**ErgoMask+Emotion**	19.9 ± 0.8	25.5 ± 1.2	32.7 ± 1.9	43.3 ± 2.1	52.6 ± 2.4	38.1 ± 1.5	34.8 ± 1.9			
Tins (s)	**ErgoMask**	1.18 ± 0.12	1.01 ± 0.05	0.79 ± 0.04	0.70 ± 0.03	0.56 ± 0.03	0.73 ± 0.03	0.76 ± 0.04	0.717	0.654	0.056
	**ErgoMask+FFP2**	1.38 ± 0.12	1.22 ± 0.09	1.03 ± 0.07	0.91 ± 0.06	0.64 ± 0.04	0.86 ± 0.04	0.88 ± 0.04			
	**ErgoMask+Emotion**	1.21 ± 0.06	1.13 ± 0.06	0.96 ± 0.05	0.75 ± 0.05	0.59 ± 0.03	0.73 ± 0.03	0.81 ± 0.04			
Texp (s)	**ErgoMask**	1.87 ± 0.15	1.26 ± 0.10	0.84 ± 0.05	0.67 ± 0.04	0.56 ± 0.02	0.87 ± 0.06	0.98 ± 0.07	1.420	0.251	0.106
	**ErgoMask+FFP2**	2.35 ± 0.22	1.45 ± 0.10	0.99 ± 0.09	0.76 ± 0.05	0.58 ± 0.02	0.95 ± 0.04	0.98 ± 0.05			
	**ErgoMask+Emotion**	1.94 ± 0.15	1.37 ± 0.07	0.95 ± 0.06	0.68 ± 0.03	0.58 ± 0.03	0.88 ± 0.04	0.98 ± 0.07			
Breathing reserve (%)	**ErgoMask**	89.2 ± 0.9	82.4 ± 1.2	60.3 ± 1.7	38.5 ± 3.3	20.4 ± 2.4	52.1 ± 3.4	62.9 ± 3.0	1.050	0.408	0.087
	**ErgoMask+FFP2**	88.8 ± 0.6	79.6 ± 1.1	60.3 ± 2.1	40.8 ± 2.4	25.5 ± 4.4	52.8 ± 2.6	63.1 ± 2.8			
	**ErgoMask+Emotion**	90.8 ± 0.7	84.2 ± 0.7	66.1 ± 1.4	44.8 ± 2.2	27.2 ± 4.2	55.0 ± 2.4	67.9 ± 2.1			

HR (ppm)	**ErgoMask**	67 ± 4.5	107 ± 3	150 ± 4	175 ± 3	178 ± 5	130 ± 5	117 ± 4			
	**ErgoMask+FFP2**	90 ± 4.3	109 ± 3	146 ± 3	170 ± 4	178 ± 3	132 ± 5	117 ± 5	2.54	0.059	0.175
	**ErgoMask+Emotion**	69 ± 8.8	105 ± 2	146 ± 3	171 ± 3	180 ± 2	132 ± 4	119 ± 4			
Time to exhaustion (s)	**ErgoMask**	-	-	335.4 ± 13.3	543.8 ± 15.9	685.9 ± 15.4	-	-			
	**ErgoMask+FFP2**	-	-	313.1 ± 5.8	485.4 ± 14.6	678.7 ± 17.0	-	-	2.559	0.094	0.176
	**ErgoMask+Emotion**	-	-	320.0 ± 7.8	527.7 ± 14.5	712.5 ± 12.3	-	-			
Speed (km/h)	**ErgoMask**	-	-	9.4 ± 0.2	12.8 ± 0.3	15.0 ± 0.3	-	-			
	**ErgoMask+FFP2**	-	-	9.0 ± 0.1	11.9 ± 0.2	14.8 ± 0.3	-	-	1.867	0.132	0.135
	**ErgoMask+Emotion**	-	-	9.2 ± 0.1	12.6 ± 0.2	15.1 ± 0.3	-	-			
VAS VE (0-10)	**ErgoMask**	8.7 ± 0.4	8 ± 0.6	-	-	5.3 ± 0.8	-	8 ± 0.5			
	**ErgoMask+FFP2**	5.8 ± 0.8	5.9 ± 0.6	-	-	4.3 ± 0.9	-	5.9 ± 0.7	1.211	0.320	0.108
	**ErgoMask+Emotion**	7.9 ± 0.3	7.2 ± 0.4	-	-	5.3 ± 0.8	-	6.9 ± 0.5			
RPE (0-10)	**ErgoMask**	0.3 ± 0.2	0.9 ± 0.3	-	-	7.9 ± 0.5	-	2.6 ± 0.5			
	**ErgoMask+FFP2**	0.3 ± 0.1	1.3 ± 0.3	-	-	8.0 ± 0.3	-	2.7 ± 0.6	0.337	0.915	0.033
	**ErgoMask+Emotion**	0.2 ± 0.1	1.2 ± 0.3	-	-	8.0 ± 0.4	-	2.8 ± 0.5			

Note. ErgoMask: Ergospirometry Mask; ErgoMask+FFP2: Ergospirometry Mask + FFP2; ErgoMask+Emotion: Ergospirometry Mask + Emotion; VE: Ventilation; VO_2: Oxygen consumption; VCO_2: Carbon dioxide production; RER: Respiratory exchange ratio; Tins: Inspiratory time; Texp: Expiratory time; HR: Heart rate; VAS VE: Ventilation visual analogue scale; RPE: Rate of perceived exertion.
